# Construction and evaluation of an antibody phage display library targeting heparan sulfate

**DOI:** 10.1007/s10719-020-09925-z

**Published:** 2020-05-28

**Authors:** Lars A.A. Damen, Els M.A. van de Westerlo, Elly M.M. Versteeg, Thierry van Wessel, Willeke F. Daamen, Toin H. van Kuppevelt

**Affiliations:** Department of Biochemistry, Radboud Institute for Molecular Life Sciences, Radboud university medical center, PO Box 9101, 6500 HB Nijmegen, the Netherlands

**Keywords:** Heparan sulfate, Antibodies, Phage display library, Consensus site, Epitope

## Abstract

Heparan sulfate (HS) is a linear polysaccharide with high structural diversity. Different HS epitopes have been detected and localized using single chain variable fragment (scFv) antibodies from a ‘single pot’ phage display library containing a randomized complementarity determining region of the heavy chain (CDR3). In this study, we created a new library containing anti-HS scFvs that all harbor a dp-38 heavy chain segment where the CDR3 region was engineered to contain the XBBXBX heparin binding consensus site (X = any amino acid, B = R, K or H). The library contained ~1.73 × 10^6^ unique antibodies and was biopanned against HS from several sources. The selected antibodies were sequenced and chemically/immunohistologically characterized. A number of 67 anti-HS scFv antibodies were selected, of which 31 contained a XBBXBX CDR3 sequence. There was a clear preference for glycine at the first and proline at the fourth position of the CDR3. The sequence GZZP(R/K)X (Z = R, K or H, but may also contain N, S, or Q) was unusually overrepresented. Selected antibodies reacted with HS/heparin, but not with other glycosaminoglycans. Antibodies reacted differentially with respect to N-, 2-O, or 6-O-desulfated heparin preparations, and showed distinct topologies of HS epitopes in rat kidney sections. The library may be instrumental in the selection of a large pool of HS epitope-specific antibodies, and - since all antibodies differ only in their 6 amino acid CDR region - may be a tool for a rational design of antibodies recognizing specific HS sulfation patterns.

**Electronic supplementary material:**

The online version of this article (10.1007/s10719-020-09925-z) contains supplementary material, which is available to authorized users.

## Introduction

Heparan sulfate (HS) is a long heterogeneously sulfated glycosaminoglycan, predominantly present in the extracellular matrix and at the cell surface. During its biosynthesis, HS is modified by sulfation and epimerization reactions, resulting in a high structural and functional diversity [1, 2]. Specific sulfation motifs within HS are involved in several physiological processes like cell signaling [2, 3], blood clotting [4], and pathological processes such as tumorigenesis [5] and Alzheimer’s disease [6, 7]. However, it remains a challenge to identify and localize HS with specific structural motifs in tissues. With the development of the ‘single pot’ Nissim antibody phage-display library [8], many single chain variable fragment (scFv) antibodies have been selected against different sources of HS [9–15]. These scFv antibodies consist of the variable part of the heavy (V_H_) and light chain (V_L_) of an immunoglobulin, joined together by a linker. In the Nissim library, the V_H_ chain is variable, whereas the V_L_ is fixed [8, 16]. About 45% of the scFv antibodies selected against HS harbor a V_H_ dp-38 germline gene segment [13–15, 17–21]. The recognition of specific HS structures by antibodies from this library is primarily generated by the V_H_ chain, especially the complementarity determining region 3 (CDR3) [8, 22]. The CDR3 is the region with most conformational variability and flexibility and is thought to have a major influence in epitope binding [11–14, 23, 24]. About 17% of the anti-HS antibodies published contain a heparin binding consensus site in the CDR3 region of the V_H_ chain [13–15, 17–21]. Heparin binding consensus sites include XBBXBX, XBBBXXBX and XBBBXXBBBXXBBX amino acid sequences where ‘B’ is a basic and ‘X’ is a random amino acid residue [13, 25, 26]. These sequences are found in several, but not all, HS-binding proteins [25–28].

To expand the number of antibodies against HS, we here engineered and evaluated an antibody phage display library targeted for HS with a single V_H_ germline segment (dp-38) and a CDR3 consisting of an XBBXBX heparin binding consensus site. As a template we used a previously obtained scFv antibody (HS4C3) which harbors both a dp-38 germline gene segment and a CDR3 sequence containing an XBBXBX heparin binding consensus site (GRRLKD) [11, 13].

## Materials & methods

### Construction of an antibody phage display library with a XBBXBX CDR3 sequence

**Fig. 1:**
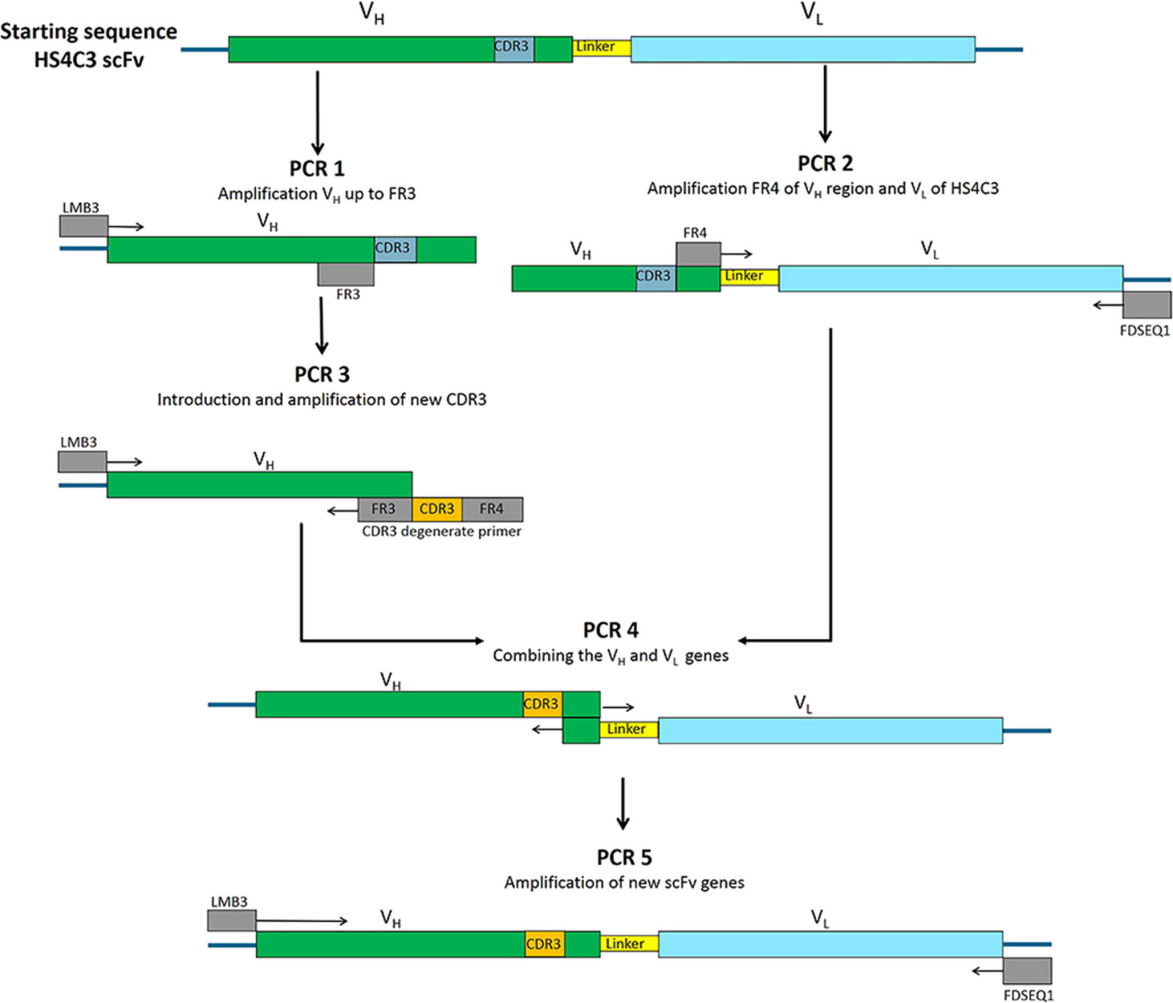
Schematic overview of PCR reactions used to construct the XBBXBX CDR3 antibody library. In PCR 1, the heavy variable chain (V_H_) up to framework 3 (FR3) of the HS4C3 coding sequence was amplified. In PCR 2, framework 4 (FR4) of V_H_ and the light variable chain (V_L_) of the HS4C3 coding sequence was amplified. The XBBXBX complementarity determining region 3 (CDR3) was engineered using the degenerate oligonucleotide CDR3 primer in PCR 3. The new V_H_ genes were combined with the V_L_ gene in PCR 4 and subsequently amplified in PCR 5. Note that the CDR3 primer contains, next to the CDR3 region, the FR4 region and part of the FR3 region. For primers, see Table 1. scFv: single chain variable fragment antibody.

The phage display library was constructed to contain only scFv antibodies harboring the V_H_3 dp-38 germline with an XBBXBX CDR3 amino acid sequence. Using the well-characterized HS4C3 scFv antibody from the Nissim library as template, the library was constructed in a series of 5 PCR procedures (PCR 1-PCR 5) (Fig. 1) [8, 13]. In all procedures, a general PCR mixture was used containing 50 mM KCl, 10 mM Tris-HCl (pH 9.0), 0.1% Triton X-100, 1.5 mM MgCl_2_, 0.5 μM primers (Table 1), 2.5 units Taq polymerase (Promega), and 0.75–1 mM dNTP in a total volume of 50 μL. The cycling temperatures were 5 min 95 °C followed by 25–40 cycles of 1 min at 95 °C, 1 min at 46–50 °C for annealing and 74 °C for 1.5–2 min for extension. Detailed PCR procedures are listed in Supplementary Table 1. In PCR 1, the V_H_ (without the CDR3 and framework 4) of the HS4C3 gene was amplified. In PCR 2, the V_L_ (including the c-Myc tag), the linker sequence and the framework 4 of the V_H_ of the HS4C3 gene were amplified. The PCR 3 procedure was performed to introduce a new CDR3 into the V_H_ gene using an 87 nucleotide degenerate reverse primer. This primer contained the CDR3 encoded as 5’-SNN NYK SNN NYK NYK SNN -3′, where S encodes for G or C; Y for C or T; K for G or T; and N for A, C, G, or T, to obtain the XBBXBX sequence (Table 1). Next, we combined the new V_H_ genes and the V_L_ gene in PCR4 using overlapping sequences (FR4 of the V_H_). The product of PCR 4 was evaluated on 1% agarose gel and isolated using the QiaEX II agarose gel extraction kit (Qiagen GmbH, Hilden, Germany). The purified product was amplified in PCR 5. The final product was purified after 1% agarose gel electrophoresis using phenol/chloroform extraction. DNA was precipitated using ethanol, the pellet was washed with 70% ethanol and dissolved in 50 μL water. The final product was digested using *NcoI* and *NotI* restriction enzymes (Life Technologies BV, Breda, the Netherlands) and ligated overnight at 16 °C into a *NcoI* and *NotI* digested pHEN1 vector (a kind gift of prof. G. Winter, Cambridge) using the Sureclone Ligation Kit (Amersham Pharmacia Biotech AB, Uppsala, Sweden). The ligation product was purified using phenol/chloroform extraction. The ligation product was transformed via electroporation into *E. coli* TG1 (K12, *sup*E, *hsd*Δ5, *thi,* Δ(*lac-pro*AB, F′(*tra*D36, *pro*AB^+^, *lac*I^q^, *lac*-ZΔM15) electroporation competent cells, efficiency ≥1.0*10^10^ cfu/μg (Stratagene Cloning Systems, La Jolla, USA). To calculate the titer of transformants, dilutions of the original electroporated bacteria were plated onto 2xTY agar plates containing 0.1% (*w*/*v*) ampicillin and 1% (w/v) glucose and incubated overnight at 37 °C.

**Table 1:** Oligonucleotide sequences used for construction of the XBBXBX CDR3 phage display library and sequencing of scFv antibody genes

Primer name	Sequence
LMB 3	5′- CAG GAA ACA GCT ATG AC -3′
FR3	5′- TCT TGC ACA GTA ATA CAC GGC CGT GTC -3’
FDSEQ1	5′- GAA TTT TCT GTA TGA GG −3′
FR4	5′- TGG GGC CAA GGT ACC CTG GTC ACC GTC TCG AGA GGT GGA GGC -3′
CDR3 primer	5′- GCC TCC ACC TCT CGA GAC GGT GAC CAG GGT ACC TTG GCC CCA SNN NYK SNN NYK NYK SNN TCT TGC ACA GTA ATA CAG GGC CGT GTC -3′ ^a^
Forlinkseq	5′- GCC ACC TCC GCC TGA ACC -3′
VL3	5′- CTT GGC ATG TGA TCC TGA −3′
PelB	5′- CCG CTG GAT TGT TAT TAC TC -3’

^a^S = [G,C], N = [A,G,C,T], Y = [C,T], K = [G,T]

### Random selection of clones

The library was plated on large 2xTY agar plates containing 0.1% (w/v) ampicillin and 1% (w/v) glucose and incubated overnight at 37 °C. Subsequently, 240 clones were randomly picked from the plate and cultured overnight in LB medium containing 1% (w/v) glucose and 0.1% (w/v) ampicillin. Plasmid DNA was collected using the QIAprep spin Miniprep Kit (Qiagen, Leusden, the Netherlands) and the V_H_ and CDR3 was sequenced using the PelB, forlinkseq and VL3 primer (Table 1).

### Selection of new scFv antibodies

Phage display biopanning procedures was performed as described previously [10]. Tubes were coated by incubation with 10 μg/mL HS from bovine kidney (Seikagaku Kogyo Co, Tokyo, Japan), HS from intestinal mucosa (Sigma), or HS isolated from human lung [17], all in 90% (*w*/*v*) (NH_4_)_2_SO_4_ [29]. Phages were produced from the library by addition of helper phage VCS-M13 (Stratagene, La Jolla, CA). Phages were precipitated and isolated using 20% (w/v) PEG/2.5 M NaCl solution and added to the coated tubes. Phages were allowed to bind for 2 h under rotation. Non-bound phages were washed away, and bound phages were eluted with 1 mL 100 mM triethylamine for 10 min. In some cases, the 10 min step was followed by an additional 20 min elution step with 1 mL 100 mM triethylamine, to retrieve phages that remained bound in the first elution step. The triethylamine was neutralized by addition of 0.5 mL 1 M Tris-HCl (pH 7.3). TG1 cells were infected with the collected phages and plated onto 2xTY agar plates containing 0.1% (*w*/*v*) ampicillin.

### Initial screening for unique antibodies against HS

Screening of scFv was performed as described previously [10, 13]. A 96-wells flat-bottom polystyrene plate was incubated overnight with 100 μL of 90% (*w*/*v*) (NH_4_)_2_SO_4_ containing 10 μg/mL HS from bovine kidney (Seikagaku), HS from porcine intestinal mucosa, heparin from porcine intestinal mucosa, dermatan sulfate from pig skin, chondroitin sulfate A (CSA) from bovine trachea, chondroitin sulfate C (CSC) from shark cartilage, thymus DNA, and hyaluronic acid from rooster comb (all from Sigma, St. Louis, MO). After washing, plates were blocked with 3% (*w*/*v*) bovine serum albumin (BSA) in 0.01 M phosphate buffered saline pH 7.2 (PBS) with 1% (*v*/v) Tween 20 for 60 min (200 μL/well). After induction of picked clones in 200 μL 2xTY with 0.1% (*w*/*v*) glucose and 0.1% (w/v) ampicillin in a 96-wells polystyrene plate, plates were centrifuged and 50 μL of the supernatant was mixed with PBS with 0.1% (v/v) Tween 20 (PBST) containing 2% (w/v) BSA in each well and incubated for 2 h. Bound antibodies were detected using a mouse monoclonal antibody against the c-Myc tag (9E10), followed by a goat-anti-mouse IgG conjugated with alkaline phosphatase (Dako Agilent, Santa Clara, CA) in 1% (*w*/*v*) BSA/PBST. Enzyme activity was detected by addition of 100 μL of 1 mg/mL p-4-nitrophenylphosphate in 1 mM diethanolamine, with 0.5 mM MgCl_2_, pH 9.8. Absorbance was measured at 405 nm. Positive clones were inoculated in 15 mL LB-medium (0.1% (w/v) ampicillin and 1% (*w*/*v*) glucose) and cultured overnight at 37 °C. Plasmid DNA was collected using the QIAprep spin Miniprep Kit (Qiagen, Leusden, the Netherlands) and the CDR3 was sequenced using the PelB, forlinkseq and VL3 primers (Table 1).

### Large-scale antibody production

For further characterization of the antibodies, scFv were obtained from the periplasmic fraction as described [13]. Bacteria were grown overnight at 37 °C in 15 mL LB-medium (100 μg/mL ampicillin and 1% (*w*/*v*) glucose). Subsequently, 10 mL of the overnight culture was added to 1 L induction medium (2xTY medium containing 0.1% (w/v) glucose and 0.1% (w/v) ampicillin). At an OD_600_ of 0.6, the antibody expression was induced by addition of isopropyl-β-D-thiogalactopyranoside (IPTG, UBPBio, Oxfordshire, UK) in a final concentration of 1 mM, and incubated for three h at 30 °C. The cultures were centrifugated for 15 min at 3561 g to separate bacteria from culture medium. Bacterial pellet was resuspended and incubated for 15 min on ice in 10 mL 0.2 mM sodium borate buffer (pH 8.0) containing 0.16 M NaCl to accommodate osmotic shock. This buffer was enriched with protease inhibitors (10 mM N-ethylmaleimide, 100 mM 6-amino hexane acid, 10 mM benzamidine-HCl, 5 mM iodoacetamide, 0.15 μM pepstatin, 30 nM apoprotein, and 100 μM phenylmethylsulfonyl fluoride), supplemented with 1% (*w*/*v*) NaN_3_. After centrifugation for 15 min at 10,000 g at 4 °C, supernatant was passed over a PBS wetted 0.45 μm filter and subsequently dialyzed against PBS, using a Visking 12-14 kDa MWCO dialysis hose (VWR, Amsterdam, NL).

### Characterization of scFv antibodies

*ELISA.* Epitope specificity of the scFv antibodies was further analyzed using an ELISA setting. The wells of a 96-wells flat bottom polystyrene microplate (Greiner Bio-One, Alphen aan de Rijn, the Netherlands) were coated by incubating with 100 μL of 90% (*w*/*v*) (NH_4_)_2_SO_4_ containing 10 μg/mL heparin, N-desulfated/N-acetylated heparin, 2*-O* desulfated heparin or 6*-O* desulfated heparin (kind gifts of dr. A. Naggi and prof. Casu of the G. Ronzoni Institute for Chemical and Biochemical Research, Milan, Italy [30, 31]). Composition of the modified heparins are listed in Supplementary Table 2 [31]. Plates were washed with PBST and blocked with 3% (w/v) BSA with 1% (*v*/v) Tween 20 in PBS. Periplasmic fractions containing the scFv antibodies were diluted 1:1 in 1% (*w*/*v*) BSA/PBST and incubated for 1.5 h. Bound scFv antibodies were detected as described above, using mouse anti-c-Myc antibody 9E10 and alkaline phosphatase conjugated goat anti-mouse antibodies, and visualized using alkaline phosphatase substrate disodium p-4-nitrophenylphosphate.

*Immunohistochemistry.* Rat kidney specimens (Wistar, male) were snap-frozen in liquid isopentane and stored at −80 °C. Cryo sections (5 μm) were rehydrated and blocked in 1% (*w*/*v*) BSA/PBST for 60 min and incubated with selected scFv antibodies in 1% (*w*/*v*) BSA/PBST for 90 min, with antibody HS4C3 as a positive control. ScFvs were detected by a 45 min incubation with mouse monoclonal anti c-Myc antibody 9E10 (1:10) in 1% (w/v) BSA/PBST, followed by a 45 min incubation with goat anti-mouse IgG, Alexa 448 conjugated (Molecular Probes Inc.) (1:300) in 1% (w/v) BSA/PBST. After each incubation, sections were washed in PBST (3 × 5 min). Sections were fixated in 100% ethanol, air-dried and embedded in 10% (w/v) Mowiol in 0.1 M Tris-HCl, pH 8.5, 25% (*v*/v) glycerol and 2.5% (w/v) NaN_3_. Sections were examined using a Leica DM 6000 fluorescence microscope and images were obtained using the Leica Microsystems software.

## Results

### Library

The library was constructed using a degenerated primer, aimed to introduce a new CDR3 consisting of an XBBXBX heparin binding consensus sequence. The primer design was such that there is a 62.5% chance of a basic amino acid (R, K or H) at the ‘B’ site, the other possibilities being Q, S or N. The latter amino acids are also frequently involved in the binding of heparin/HS [25, 32]. The theoretical diversity of the library is 1.34 × 10^8^ unique clones, encoding for 1.73 × 10^6^ unique full-length proteins. To obtain an impression of the actual diversity of the library, 222 randomly picked clones were sequenced, yielding 194 full sequences of which 189 (97.4%) were unique (Table 2, Supplementary Table 3). This indicates that the library contains about 1.31 × 10^8^ clones encoding for 1.68*10^6^ (97% of 1.73 × 10^6^) unique full-length proteins, which is 97% of the theoretical diversity. Screening of CDR3 sequences demonstrated that 23.3% (44/194) of the randomly selected clones contained a XBBXBX amino acid sequence, similar to what was theoretically estimated (24.4%) (Table 2). Selection against HS (see below) yielded 67 unique scFv antibodies (Table 3). The XBBXBX CDR3 was more abundant in the scFv selected against HS (46%, 33/67) than in randomly picked clones (23.3%) (*p* < 0.01) (Table 2). In addition, 35 of the 67 (52%) scFv antibodies selected against HS had glycine (G) as the first amino acid residue, and 30 of the 67 (44.5%) contained proline (P) as the fourth amino acid residue (Table 4). A combination of G at the first position and P at the fourth, together with a R or K at the fifth position, was observed 23 times (34.4%) (Table 2). This combination was completely absent in randomly selected clones (theoretically 0.2% of all clones harbor this sequence (Table 2)).

**Table 2:** Diversity of the XBBXBX CDR3 antibody library

	Randomly picked clones	Clones selected against heparan sulfate
Number of clones selected	222	67
Full length CDR3 V_H_ DNA sequence (% of selected clones)	194 (87.3%)	67 (100%)
Unique CDR3 V_H_ DNA sequence	189 (97.4%)	67 (100%)
XBBXBX^a^ sequence of CDR3 V_H_ (% of unique sequences)	44 (23.3%)^b^	31 (46%)^b^
GZZP(R/K)X^c^ sequence of CDR3 V_H_ (% of unique sequences)	0^d^	23 (34.3%)^d^

^a^‘B’ encodes the basic residues H, R, or K, and ‘X’ encodes any amino acid

^b^Theoretical value: 24.4%

^c^‘Z’ encodes H, R, K, N, Q, or S

^d^Theoretical value: 0.2%

**Table 3:**
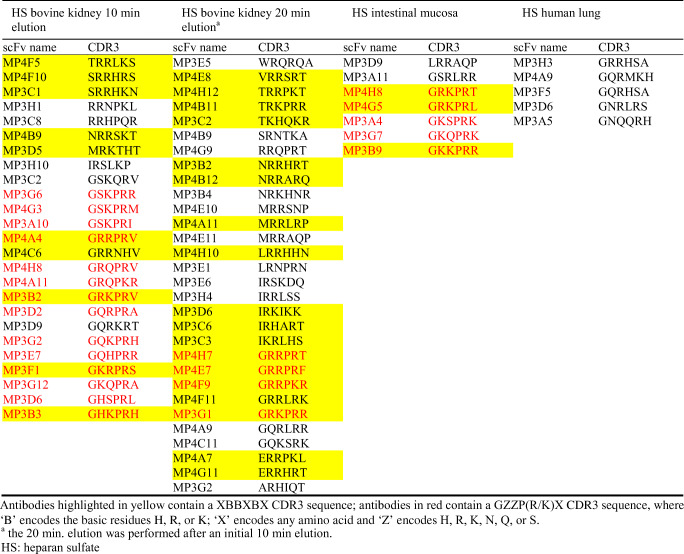
Overview of scFv antibodies selected against HS preparations using the XBBXBX CDR3 antibody library

**Table 4:** Occurrence (in %) of specific amino acids in the CDR3 of anti-HS scFv antibodies

	Position in the CDR3 V_H_					
Amino acid	X	B^a^	B^a^	X	B^a^	X
A	1.5			6.0		9.0
D						
N	6.0	3.0	4.5	1.5	6.0	4.5
R	4.5	64.0	47.5	1.5	56.5	17.8
C						
Q		12.0	10.5	4.5	7.5	3.0
E	3.0					
G	52.0					
H		3.0	7.5	12.0	6.0	6.0
I	9.0			3.0		1.5
L	4.5			13.5		6.0
K		10.5	24.0	3.0	18.0	7.5
M	6.0			1.5		
F						3.0
P				44.5		7.5
S	4.5	7.5	6.0	6.0	9.0	9.0
T	6.0			3.0		17.8
W	1.5					
Y						
V	1.5					7.5
Total	100%	100%	100%	100%	100%	100%

^a^‘B’ may contain basic residues (R,K,H), but also Q, N, or S

### Biopanning of library against various HS and characterization of antibodies

After biopanning against immobilized HS, 25 unique antibodies were selected against bovine kidney (10 min elution), and 30 after an additional 20 min elution (Table 3). Further, 5 antibodies were selected against HS from human lung, and 7 against porcine intestinal mucosa after 10 min elution (Table 3). The reactivity of all antibodies with various types of glycosaminoglycans and modified heparin preparations was determined (Supplementary Table 4 and 5), as well as the location of antibody-defined HS epitopes in the kidney (Supplementary Table 6). To illustrate the characteristics of some anti-HS antibodies, we here highlight two antibodies selected against bovine kidney after 10 min elution (MP3C1 and MP4A11’10.b.k.), two against bovine kidney after an additional 20 min elution (MP4F11 and MP3B2’20.b.k.), two against HS from porcine mucosa (MP4G5, and MP3G7), and one against human lung HS (MP3A5) (Table 3). One randomly picked scFv antibody (MPB49) that was not reactive with HS was also included. Antibody specificity was screened using ELISA. Anti-HS antibodies were moderate to strong binders to heparin (Table 5). All antibodies were moderate to strong binders to HS from bovine kidney, whereas antibodies MP4G5 and MP4F11 showed strong binding towards HS from intestinal mucosa. Antibodies were not reactive with other glycosaminoglycans (chondroitin sulfate A and C, dermatan sulfate, hyaluronic acid) and DNA, with the exception of MP4F11 which showed moderate binding to dermatan sulfate (Table 5).

**Table 5:** Reactivity of selected anti-HS scFv antibodies against several glycosaminoglycans as assessed by ELISA

	MP3C1	MP4A11^a^	MP3B2^b^	MP4F11	MP3G7	MP4G5	MP3A5	MPB49
Heparin	++	+++	+	+++	+++	+++	+	–
HS b.k.	++	+	++	++	+++	+++	+	–
HS i.m.	–	+	±	++	+	++	±	–
DS	–	–	–	+	–	–	–	–
CSA	–	–	–	–	–	–	–	–
CSC	–	–	–	–	–	–	–	–
DNA	–	–	–	–	–	–	–	–

Reactivity: +++: very strong, ++: strong, +: moderate, ±: weak, − absent

^a^Obtained from selection against HS from bovine kidney after 10 min elution (MP4A11’10.b.k)

^b^Obtained from selection against HS from bovine kidney after additional 20 min elution (MP3B2’20.b.k)

HS b.k.: heparan sulfate from bovine kidney; HS i.m.: heparan sulfate from porcine intestinal mucosa; DS: dermatan sulfate; CSA: chondroitin sulfate A; CSC: chondroitin sulfate C; DNA: deoxyribonucleic acid

### Analysis of sulfate groups in HS-epitope of scFv antibodies

Reactivity of the anti-HS antibodies was further studied using modified heparin preparations (N-desulfated/N-acetylated, 2*-O* desulfated, and 6*-O* desulfated heparin; Table 6). Reactivity of MP4F11 and MP3G7 was observed towards all desulfated forms of heparin, indicating that both antibodies recognize heparan sulfate with a moderate degree of sulfation. In contrast, MP3B2’20.b.k. did not react with the modified heparin molecules, indicating its preference for much higher sulfated forms of HS. The MP4G5 and MP3A5 antibodies did not react with 2*-O* desulfated and N-desulfated/N-acetylated forms of heparin, but were able to detect the 6*-O* desulfated form of heparin (note that in this heparin preparation about 30% of the 6*-O* sulfate groups are still present). MP4A11’10.b.k. was able to detect both 6*-O* desulfated and 2*-O* desulfated heparin, but did not bind to the N-acetylated form of heparin, indicating N- sulfation to be essential for binding.

**Table 6:** Reactivity of selected anti-HS scFv antibodies against modified heparin preparations, as assessed by ELISA

	MP3C1	MP4A11^a^	MP3B2^b^	MP4F11	MP3G7	MP4G5	MP3A5
Heparin	+	+	++	++	++	++	+
N-desulfated/N-acetylated heparin	–	–	–	+	+	–	–
2*-O* desulfated heparin	–	+	–	++	+	–	–
6*-O* desulfated heparin^c^	±	+	–	++	++	++	++

Reactivity: +++: very strong, ++: strong, +: moderate, ±: weak, − absent

^a^Obtained from selection against HS from bovine kidney after 10 min elution (MP4A11’10.b.k.)

^b^Obtained from selection against HS from bovine kidney after additional 20 min elution (MP3B2’20.b.k.)

^c^23% of 6-O sulfate still present

HS: Heparan sulfate

### Localization of HS epitopes in rat kidney sections

**Fig. 2:**
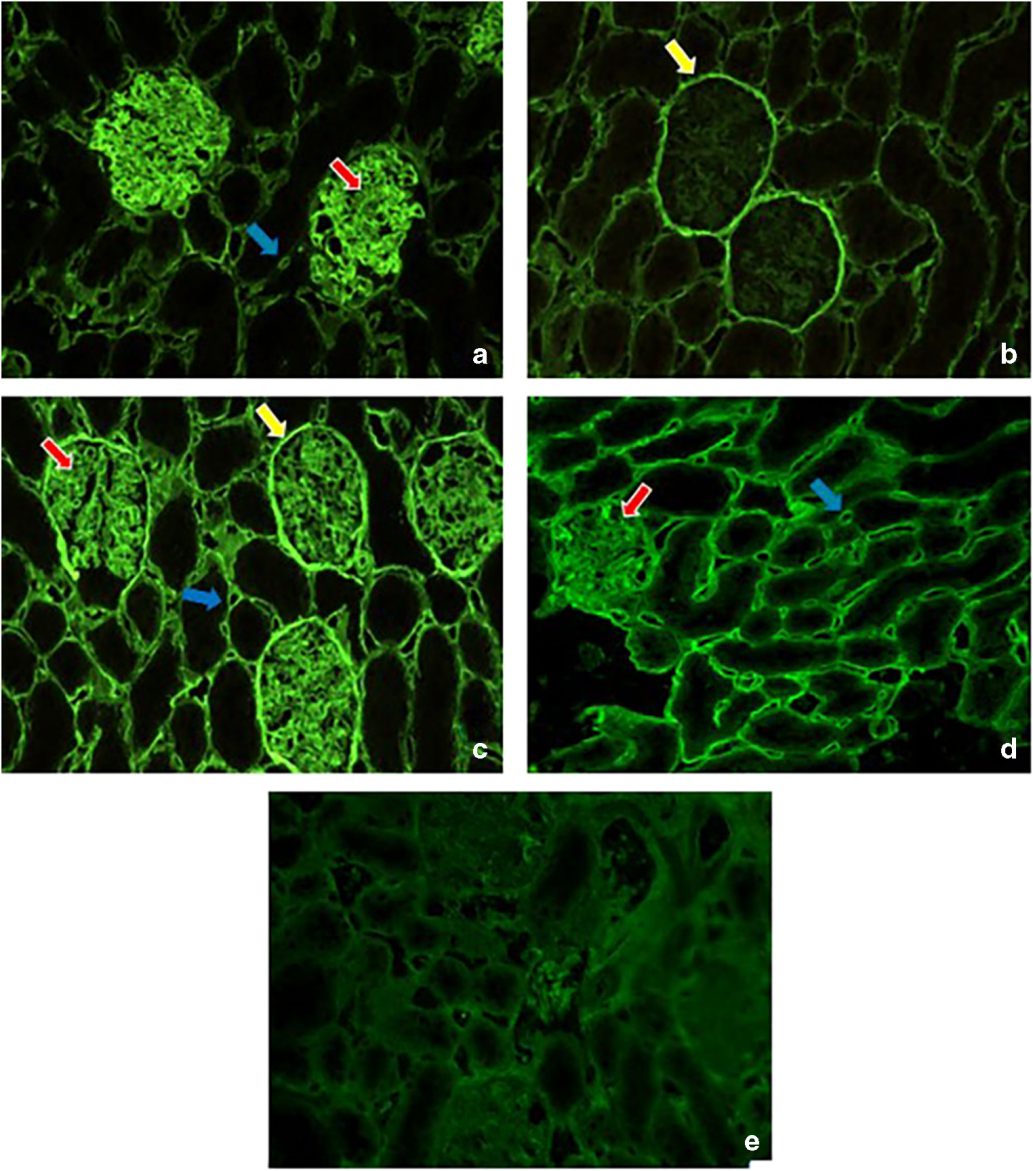
Immunofluorescent staining of rat kidney cryosections with selected anti-HS scFv antibodies indicate three distinct types of staining patterns. Staining type 1 (A, antibody MP3C1) comprises staining of the glomerulus (red/white arrow) and peritubular capillaries (blue arrow); staining type 2 (B, antibody MP4A11’10.b.k.) primarily comprises staining with Bowman’s capsule (yellow arrow); staining type 3 (C, antibody MP4F11) comprises staining of the glomerulus, the peritubular capillaries and Bowman’s capsule. Control antibody HS4C3 (D) results in type 1 staining. Antibody MPB49 (E) does not recognize a HS epitope in rat kidney sections HS: heparan sulfate.

The location of the epitope defined by the anti-HS scFv antibodies was analyzed in rat kidney cryosections and compared to the original HS4C3 antibody. Although all scFvs have a unique CDR3 sequence, three main patterns of staining were observed (Fig. 2, Table 7). MP3C1 (Fig. 2A), MP3B2’20.b.k. and MP3A5 mainly stained the glomerulus and the peritubular capillaries, while MP4A11’10.b.k. (Fig. 2B) and MP3G7 primarily recognized Bowman’s capsule. Both Bowman’s capsule and the glomerulus were recognized by MP4F11 (Fig. 2C), MP4G5 and MP4F11). Antibody MPB49 (randomly selected) was not reactive with kidney sections (Fig. 2E).

**Table 7:** Distribution of HS-epitopes in rat kidney defined by selected antibodies as assessed by immunohistochemistry

	MP3C1	MP4A11^a^	MP3B2^b^	MP4F11	MP3G7	MP4G5	MP3A5	HS4C3
Glomerular capillary tuft	++	–	++	++	±	+	++	++
Glomerular mesangium	+	–	ND	+	+	+	±	+
Bowman’s capsule	–	++	–	+	++	+	–	–
Peritubular capillaries	+	±	+	+	+	+	±	+

Staining: ++: strong, +: moderate, ±: weak, − absent

^a^Obtained from selection against HS from bovine kidney after 10 min elution

^b^Obtained from selection against HS from bovine kidney after additional 20 min elution

ND: no data; HS: heparan sulfate

## Discussion

### Library development

In this study, we successfully developed a new antibody phage display library designed to target HS by engineering the CDR3 in the V_H_ of the antibodies. The CDR3 is thought to be the largest contributor to antigen recognition and in this study we designed it to contain a heparin binding consensus site [33]. In proteins, XBBXBX, XBBBXXBX, and XBBBXXBBBXXBBX sequences have been described as heparin binding consensus sites [26]. We selected the XBBXBX sequence, since a number of the previously obtained anti-HS scFvs contain short (<10 amino acids) CDR3 sequences, which may be favorable for binding [13, 14, 34]. To engineer the XBBXBX CDR3, we used a degenerate primer, which also allowed S, N, and Q in the ‘B’ positions. As a result, 23.5% of our library contained the actual XBBXBX amino acid sequence in the CDR3. The XBBXBX consensus site is not essential for protein-HS interaction, although many proteins use it [28, 35]. Of the selected anti-HS antibodies 46% contained a XBBXBX CDR3 sequence. The presence of S, N, or Q at the ‘B’ positions of the CDR3 was found in a number of anti-HS scFv antibodies indicating that the XBBXBX is not an absolute requirement for binding. Interestingly, these residues have been described in heparin-binding peptides [25], and a number of previously obtained anti-HS scFv antibodies contain these residues within their CDR3 [12–15].

When evaluating the ‘X’ residues within the XBBXBX CDR3 of anti-HS scFvs, an abundance of G and P at the first and fourth position respectively, was noticed. These residues have been described to provide structural stability to a loop structure in the CDR3 [36]. Moreover, these residues have been frequently found in heparin-binding peptides alongside R, K, H, S, N, and Q [25]. Anti-HS scFv antibodies from the library seem to have a preference for the GZZP(R/K)X sequence, where ‘Z’ encodes R, K, H, S, N, or Q. Since the amino acids G and P are frequently found in turns, bends and folds [37–40] the GZZP(R/K)X sequence may form a charged pocked structure that binds to a specific monosaccharide or a specific spatial orientation of sulfate groups, which are generally present on the outside of a HS molecule. The G and P would then contribute to the formation of a pocket, and the positively charged amino acids would confer binding to the negatively charged saccharide.

### Antibody characterization

From the library, 67 new scFv antibodies against HS were selected, indicating the versatility of the library. The biosynthesis of HS allows for a large number of motifs and patterns within the molecule. The initial HS backbone consists of repeating disaccharide units composed of a glucuronic acid and an N-acetyl glucosamine. The acetyl group of the N-acetyl glucosamine can be exchanged for a sulfate group, initiating further modifications including epimerization of a glucuronic acid into an iduronic acid, the addition of a sulfate group to the C2 hydroxyl group of the uronic acid, and the addition of sulfates to the C6 and C3 hydroxyl groups of the glucosamine. Since these modifications occur in clustered regions, HS consists of low and highly sulfated domains. As a result, HS has a large structural variability and many different sulfation patterns are possible [1, 2, 41]. With the new library, we aimed to obtain scFv antibodies directed against the different HS epitopes. We analyzed the reactivity of 67 anti-HS scFv antibodies with modified heparin preparations and studied the HS epitope in rat kidney sections. A number of antibodies had a distinct reaction profile. For instance, antibody MP3B2’20.b.k. did not react with N, 2*-O* or 6*-O* desulfated heparin preparations and strongly stained the kidney glomerulus, but not Bowman’s capsule. On the other hand, antibody MP4A11 reacted with 2*-O*, and 6*-O* desulfated heparin, but not with N-desulfated (N-acetylated) heparin, and stained Bowman’s capsule, but not the glomerulus. Antibody MP4F11 reacted with all desulfated heparin preparations and stained the glomerulus as well as Bowman’s capsule. This suggests that *e.g*. antibody MP3B2’20.b.k. recognizes a highly sulfated HS domain, whereas antibody MPF11 recognizes a low sulfated region. Clearly, more in depth studies are needed to specify the exact nature of the HS structure(s) recognized by the antibodies. New developments in HS microarrays and cell-based HS libraries offer potential to do such analyses [42–44].

### Anti-HS scFvs on design

In this study we aimed to obtain new anti-HS scFv directed towards different HS-epitopes by engineering the CDR3. Since this approach was successful, applying specific modifications to the anti-HS scFvs may prove to be effective to obtain antibodies against specific sulfation patterns. These modifications may not be limited towards the CDR3, since regions located outside the linear heparin binding site have been demonstrated to influence HS binding [45–47]. Knowledge on the specific amino acids involved in HS binding may allow for a rational design of antibodies targeting specific sulfation patterns. Strategies such as “protect and label” can identify essential amino acids involved in HS binding [48]. Once identified, site-directed mutagenesis of the CDR3 and other regions within V_H_ and/or V_L_ may result in the generation of anti-HS scFvs recognizing specific sulfation patterns. These tools may be instrumental in elucidating of the function of HS sulfation patterns in (patho)physiological processes.

In conclusion, we successfully constructed a new anti-HS scFv library, engineering the CDR3 to contain the heparin-binding consensus site XBBXBX. The library may be a rich source of new antibodies directed against different HS-epitope, and may allow the design of novel antibodies recognizing specific sulfation patterns.

## Electronic supplementary material

ESM 1 (PDF 238 kb)

ESM 2 (DOCX 12.2 kb)

## Abbreviations

BSA: Bovine serum albumin
CDR3: Complementarity determining region 3 of heavy variable chain
CSA: Chondroitin sulfate A
CSC: Chondroitin sulfate C
DNA: Deoxyribonucleic acid
DS: Dermatan sulfate
FR3: Framework 3 of heavy variable chain
FR4: Frame work 4 of heavy variable chain
HS: Heparan sulfate
PBS: phosphate buffered saline
scFv: Single chain variable fragment
V_H_: Heavy variable chain
V_L_: Light variable chain
