# Supplementary material for: Construction and evaluation of an antibody phage display library targeting heparan sulfate
Source: Glycoconj J. 2020 May 28;37(4):445–55. doi: 10.1007/s10719-020-09925-z (PMC7329785; doi:10.1007/s10719-020-09925-z)
Supplement: Supplementary file 1 — (PDF 238 kb) [file 10719_2020_9925_MOESM1_ESM.pdf]

Construction and evaluation of an antibody phage display library targeting heparan sulfate

Lars A.A. Damen[1], Els M.A. van de Westerlo[1], Elly M.M. Versteeg[1], Thierry van Wessel[1], Willeke F. Daamen[1], Toin H. van Kuppevelt[1]*

[1] Department of Biochemistry, Radboud Institute for Molecular Life Sciences, Radboud university medical center, PO Box 9101, 6500 HB Nijmegen, the Netherlands

* The author to whom correspondence should be addressed: Tel: +31-24-3616759; e-mail: Toin.vanKuppevelt@radboudumc.nl

Supplementary Table 1: PCR procedure, materials and conditions used for construction of the XBBXBX CDR3 library

PCR	PCR template	Template amount	Forward primer	Reverse primer	Cycles	Annealing temp (°C)	Extension time (min)	dNTP conc. (mM)
1	pHEN1 HS4C3	20 ng	LMB3	FR3	25	50	1.5	0.75
2	pHEN1 HS4C3	20 ng	FR4	FDSEQ1	25	46	1.5	0.75
3	PCR product 1	21 µL	LMB3	CDR3 primer	30	50	2	1
4	PCR product 2&3	17 µL	-	-	40	50	2	0.75
5	PCR product 4	17 µL	LMB3	FDSEQ1	40	50	2	0.75

Supplementary Table 2: Composition of modified heparin molecules (taken from [31]).

**Molecule**	**Characteristics**
Heparin	11.3% N-acetyl, 88.7% N-sulfate, 69% 2-*O* sulfate, 79% 6-*O* sulfate
N-desulfated/N-acetylated heparin	100% N-acetyl, 0% N-sulfate, 69% 2-*O* sulfate, 79% 6-*O* sulfate
2-*O* desulfated heparin	13% N-acetyl, 87% N-sulfate, 0% 2-*O* sulfate, 79% 6-*O* sulfate
6-*O* desulfated heparin	13% N-acetyl, 87% N-sulfate, 67% 2-*O* sulfate, 23% 6-*O* sulfate

Supplementary Table 3: Overview of amino acid and nucleotide sequence of the heavy chain CDR3 of randomly picked clones from the XBBXBX CDR3 antibody library

#	ID	CDR3	CDR3 sequence
1	MPB65	YSNRHA	TACAGCAACCGGCATGCT
2	EW125	YSHPKA	TACAGCCACCCCAAGGCC
3	MPB36	YRSEQD	TACCGCAGCGAGCAGGAC
4	EW151	YRRTKT	TACCGTCGAACCAAAACG
5	EW112	YRNRNV	TACCGGAACAGGAACGTC
6	EW028	YRNLKP	TACCGAAACCTCAAGCCA
7	MPB25	YQQNQR	TACCAACAAAACCAACGC
8	EW038	YNRDHY	TACAACAGAGACCACTAC
9	MPB49	WNRDRQ	TGGCGCAACGACCGCCAG
10	EW003	WKSRRT	TGGAAGAGTCGCCGTACA
11	MPB28	VQRPKT	GTGCAACGGCCCAAGACC
12	EW085	VNQTRR	GTGAACCAGACCCGCCGC
13	MPB03	TSRNHI	ACAAGCAGGAACCACATC
14	EW122	TRRSRC	ACCAGGCGTAGCAGATGC
15	EW133	TRQTIP	ACCCGCCAAACCATACCC
16	EW034	TRNPRP	ACGCGTAACCCCCGCCCC
17	EW035	TRNPRP	ACGCGTAACCCCCGCCCC
18	EW070	TRNCNL	ACCAGGAACTGCAATCTC
19	MPB40	TRHRKY	ACCCGACACAGGAAGTAC
20	MPB29	TRH*SC	ACGCGGCACTAGAGCTGC
21	MPB71	TNRVKR	ACCAACCGTGTGAAGCGC
22	EW071	TNNENN	ACCAACAACGAGAATAAC
23	EW101	TKRSSN	ACCAAGCGTTCCAGCAAC
24	EW067	TKRPQS	ACCAAGCGCCCGCAGTCG
25	EW126	TKNHSR	ACCAAAAACCACAGCAGG
26	EW152	TKHSQA	ACCAAGCACTCCCAAGCC
27	MPB20	THRYHE	ACGCACCGATACCACGAG
28	MPB11	THQMRP	ACGCATCAAATGCGTCCG
29	MPB35	THQCKY	ACCCACCAGTGCAAATAC
30	EW029	THKRRD	ACCCACAAACGCCGCGAC
31	EW144	SSHDK*	AGCAGCCATGACAAGTAG
32	EW018	SRRSRT	TCCCGCCGGAGCCGCACG
33	EW053	SRRSKT	TCGCGTCGGTCGAAAACG
34	MPB54	SRRKKR	AGCCGCCGGAAGAAACGT
35	MPB58	SRRAKY	TCCCGAAGAGCCAAATAC
36	EW020	SRRAKT	AGCCGCAGAGCCAAAACC
37	MPB16	SRRAHT	TCGCGCCGGGCGCATACC
38	EW060	SRQHRT	AGCCGCCAACACCGCACC
39	EW146	SRQFRP	TCCAGGCAGTTCCGCCCC
40	EW050	SRKHHA	TCCCGAAAGCACCATGCT
41	EW066	SRH*NP	AGCCGGCACTAGAACCCC
42	EW001	SRHERR	AGCCGACATGAGCGCAGG
43	EW037	SQQLRT	AGCCAACAACTCCGCACC
44	MPB39	SKSPNT	TCCAAAAGCCCGAACACT
45	EW075	SKNTNC	AGCAAAAATACGAATTGC
46	MPB10	SHNTQT	TCCCACAATACCCAAACC
47	EW108	RSRRNT	AGGAGCCGTCGCAATACC
48	EW007	RSHAR*	CGCAGCCACGCCCGTTAG
49	MPB53	RRSHQH	CGCCGCAGCCACCAGCAC
50	EW022	RRSFRA	CGCCGCAGTTTCCGTGCT
51	EW046	RRRDQK	CGCCGACGCGACCAAAAG
52	EW063	RRQSKR	CGGCGACAGAGCAAGCGT
53	MPB33	RRQINA	AGGCGACAAATCAACGCC
54	EW068	RRQCRS	CGCCGTCAGTGCCGATCC
55	EW019	RRNPRC	AGGCGCAACCCCCGCTGC
56	MPB06	RRNPQS	CGCCGCAACCCCCAATCC
57	EW006	RRKTRI	CGCCGCAAGACCCGCATC

58	MPB21	RRKRSA	CGCCGCAAGAGGAGTGCA
59	MPB17	RQRARG	AGGCAACGCGCCAGAGGT
60	EW156	RQHHHP	CGCCAGCATCACCACCCC
61	EW128	RNSVKS	CGGAACAGTGTGAAGAGC
62	MPB50	RNRPRT	CGCAACCGCCCCCGCACT
63	EW086	RNHHNT	CGCAACCACCACAATACG
64	EW064	RHRERT	AGGCATAGGGAGCGGACC
65	MPB15	RHRANS	CGGCACAGAGCCAACTCC
66	EW036	RHQANQ	CGCCACCAGGCGAACCAG
67	EW153	RHNLRH	AGGCACAACCTGAGACAC
68	EW002	RAETQM	CGGGCCGAGACCCAGATG
69	MPB44	QRSLSP	CAGCGAAGTCTCAGCCCG
70	EW096	QRRQSA	CAGCGACGCCAGAGCGCG
71	EW142	QRNPST	CAGAGAAATCCGAGCACG
72	MPB69	QRKNRP	CAGAGAAAAAACCGCCCC
73	EW056	QRHAQA	CAGCGCCACGCCCAGGCA
74	EW010	QNHRRP	CAGAATCATCGCCGCCCA
75	MPB61	QKSPQP	CAGAAAAGCCCCCAACCT
76	EW027	QKHRHH	CAGAAACACCGGCATCAT
77	MPB51	QHRKHL	CAGCACAGAAAGCACCTC
78	EW017	QHRGKM	CAGCACAGGGGCAAAATG
79	EW098	QHRDKC	CAGCATCGCGACAAATGC
80	EW072	PRSRRV	CCCCGCAGCCGCAGAGTC
81	EW111	PRRTRS	CCCAGGCGCACCCGCTCC
82	EW021	PRRTRP	CCCCGGAGAACCCGCCCA
83	MPB08	PRRSRI	CCGAGGCGTAGCCGGATC
84	EW033	PRRCSG	CCGCGGAGGTGCAGCGGG
85	MPB37	PRRART	CCGCGCCGAGCCCGCACT
86	EW119	PRNTKR	CCGCGGAATACGAAGCGC
87	MPB23	PRKNNP	CCGCGCAAAAACAACCCC
88	EW109	PQKVNT	CCCCAAAAAGTCAACACC
89	EW105	PQHFKA	CCCCAACACTTCAAAGCC
90	EW044	PNRAHN	CCGAACCGAGCCCACAAC
91	EW073	PNNRHL	CCCAACAACAGGCACCTC
92	MPB01	PKRTRN	CCCAAACGCACCCGGAAC
93	EW134	PKKPRT	CCCAAAAAGCCCCGCACG
94	EW031	PKHTNT	CCCAAACACACCAACACC
95	EW103	PIHSRT	CCCATCCACTCCCGAACC
96	EW135	PHSGSL	CCGCACAGCGGGAGCCTC
97	EW065	PHQHHA	CCCCACCAGCACCATGCC
98	EW124	PHKPNS	CCCCACAAGCCCAACTCT
99	EW127	PHKPHS	CCCCATAAACCCCACTCC
100	MPB32	P*KQRT	CCCTGAAAGCAGCGGACG
101	EW117	NRSAKC	AACCGAAGCGCCAAGTGC
102	MPB31	NRRIRN	AACAGGCGGATCAGAAAC
103	EW052	NRNPNK	AACCGCAACCCCAACAAG
104	EW136	NRNEQT	AACAGGAACGAGCAGACA
105	EW058	NRNANI	AACAGAAACGCCAACATC
106	EW137	NRHMQS	AACCGTCATATGCAGTCA
107	EW013	NQRHQA	AACCAGCGGCACCAGGCT
108	EW005	NNHLKH	AACAACCACCTCAAACAC
109	EW054	NHRQQV	AACCACAGACAGCAAGTC
110	EW147	MRRPRP	ATGCGCCGGCCCCGCCCG
111	MPB47	MHRINP	ATGCACCGCATCAACCCA
112	EW106	LSR*RT	CTCAGCCGCTAGAGGACG
113	EW140	LRSSRR	TTGCGCAGCTCCAGACGC
114	MPB56	LRNSRP	CTCAGGAATTCCCGGCCC
115	EW097	LRKSNP	TTGCGCAAATCCAACCCC
116	EW110	LRKPHL	CTGCGTAAGCCCCACCTG
117	MPB14	LQSQQV	CTCCAGAGCCAGCAAGTCC

118	EW138	LQRAR*	TTGCAACGAGCCCGCTAA
119	EW080	LNHSSD	CTGAACCATTCGAGTGAC
120	EW123	LKNEHT	CTCAAAAACGAGCATACG
121	MPB05	LHRARS	CTGCACCGAGCGCGCAGC
122	EW113	LHQPHD	CTGCACCAGCCCCACGAC
123	EW011	PKSNRV	CCRAAAAGTAACCGCGTC
124	EW012	KRHNQP	AAGCGCCATAACCAACCA
125	EW015	KNQSNT	AAGAACCAAAGCAACACC
126	EW083	KNNGHP	AAGAATAACGGGCACCCC
127	EW076	KKQAQP	AAGAAGCAAGCGCAACCG
128	EW077	KKQAQP	AAGAAGCAAGCGCAACCG
129	EW074	KKNQRR	AAGAAAAACCAGCGCCGG
130	EW100	KKKSKR	AAGAAGAAAAGCAAACGC
131	EW048	IRRAQN	ATCAGACGCGCGCAAAAC
132	EW114	IQRHHT	ATCCAGCGACACCACACC
133	EW078	IQKHKN	ATCCAAAAGCACAAAAAC
134	EW079	INQVQP	ATCAACCAAGTGCAGCCG
135	EW023	IKSHQD	ATCAAAAGCCACCAGGAC
136	MPB09	IKRSKS	ATCAAGAGATCCAAAAGC
137	EW148	IKHPQP	ATCAAGCATCCCCAGCCG
138	EW032	IHQTQT	ATCCACCAAACGCAAACC
139	EW158	IHNNNA	ATCCACAACAACAATGCC
140	EW115	HSHGSN	CACAGCCACGGCAGCAAC
141	MPB42	HRSLRA	CACAGGAGCCTGCGAGCG
142	EW154	HRSHHQ	CACAGAAGCCACCACCAG
143	EW102	HRRWHH	CACAGGCGCTGGCACCAC
144	MPB59	HRRLSP	CACCGAAGACTCAGCCCG
145	MPB27	HRRINT	CACAGGCGCATCAACACC
146	EW093	HRQTQA	CACCGACAAACCCAGGCC
147	MPB38	HRQHQE	CACCGTCAGCACCAGGAG
148	EW041	HRQDHY	CACCGGCAAGACCACTAC
149	EW039	HRNTQA	CACAGAAACACCCAAGCG
150	MPB64	HRNLQL	CACCGCAACTTGCAGCTC
151	EW059	HRNIRR	CACCGCAACATCCGACGC
152	EW107	HRKTRR	CACCGCAAAACGCGCCGT
153	MPB72	HRHQRT	CACCGCCACCAGCGTACT
154	MPB26	HQRWRS	CACCAAAGGTGGAGGTCG
155	EW042	HQRARA	CACCAACGCGCCCGCGCG
156	EW004	HQQVKR	CACCAGCAGGTGAAACGC
157	MPB45	HQQRNI	CACCAGCAAAGGAACATC
158	EW120	HQKRNQ	CACCAAAAACGGAACCAA
159	MPB46	HPRRQ	CACCCGAGGCGCCAAAG
160	EW116	*HNTQP	TAGCACAACACCCAACCC
161	MPB66	HNRLNI	CACAACCGGCTGAATATC
162	EW043	HNKARR	CACAACAAAGCCCGACGC
163	EW025	HNHNRS	CACAACCATAACCGCTCC
164	MPB48	HHHKHH	CACCACCACAAGCACCAC
165	EW160	GSRKKN	GGGAGTAGGAAGAAGAAC
166	EW057	GRRLKD	GGTAGGAGGCTTAAGGAT
167	EW069	GRRLKD	GGTAGGAGGCTTAAGGAT
168	EW145	GRRLKD	GGTAGGAGGCTTAAGGAT
169	MPB43	GRRLKD	GGTAGGAGGCTTAAGGAT
170	MPB70	GRNSHT	GGCAGGAATAGCCATACG
171	MPB19	GRKSNG	GGCCGAAAGTCCAACGGC
172	EW157	GRHRRT	GGCCGCCACCGCAGGACC
173	EW121	GKRTRV	GGCAAGAGGACGAGAGTC
174	MPB57	GHQARR	GGCCACCAAGCCCGTAGG
175	EW049	FSQDNP	TTCAGCCAAGACAACCCA
176	EW150	EHRDNS	GAGCACCGCGACAACTCC
177	MPB55	DRRPRS	GACCGCAGACCGCGGAGC

178	EW099	DQRLKS	GACCAGCGCCTGAAAAGC
179	EW051	DQK*SD	GACCAAAAATAGAGCGAC
180	EW130	DNKNHS	GACAACAAAAACCACAGT
181	EW081	DKNTHT	GACAAAAACACCCACACG
182	EW045	DHRPRC	GACCACAGACCCCGATGC
183	EW061	CHSAQR	TGCCATAGCGCGCAAAGG
184	EW094	CHQRKS	TGCCATCAGAGGAAGAGC
185	MPB22	CHQNKP	TGCCACCAGAACAAGCCG
186	EW149	ARRLQQ	GCCCGTAGACTCCAACAG
187	EW092	ARQMQS	GCCCGCCAGATGCAATCC
188	EW014	ARNTQP	GCCCGCAACACCCAACCC
189	EW141	AQNANK	GCGCAAAATGCGAATAAG
190	EW095	ANSRKG	GCGAACAGCCGCAAGGGC
191	EW024	ANRPQG	GCGAATCGACCCCAGGGA
192	EW047	ANRHKV	GCGAACAGGCACAAAGTC
193	MPB18	AKSYRY	GCCAAAAGCTACCGTTAC
194	EW026	AHRKHL	GCCCACAGAAAGCACCTG

*stop codon

Supplementary Table 4: Reactivity of scFv antibodies selected against HS towards several GAGs as assessed by ELISA.

scFv	CDR3	Panning material	HS b.k.	HS i.m.	Hep	DS	CSA	CSC	DNA	HYA
MP4F5	TRRLKS	Bovine kidney HS 10 min elution	+++	-	++	-	-	-	-	-
MP4F10	SRRHRS	Bovine kidney HS 10 min elution	±	-	++	-	-	-	-	-
MP3C1	SRRHKN	Bovine kidney HS 10 min elution	++	-	++	-	-	-	-	-
MP3H1	RRNPKL	Bovine kidney HS 10 min elution	++	-	+++	-	-	-	-	-
MP3C8	RRHPQR	Bovine kidney HS 10 min elution	++	-	++++	-	-	-	-	-
MP4B9	NRRSKT	Bovine kidney HS 10 min elution	++	-	++++	-	-	-	-	-
MP3D5	MRKTHT	Bovine kidney HS 10 min elution	+	-	+++	-	-	-	-	-
MP3H10	IRSLKP	Bovine kidney HS 10 min elution	+++	++	++++	+	-	-	-	-
MP3C2	GSKQRV	Bovine kidney HS 10 min elution	+	-	+++	-	-	-	-	-
MP3G6	GSKPRR	Bovine kidney HS 10 min elution	++	-	++++	-	-	-	-	-
MP4G3	GSKPRM	Bovine kidney HS 10 min elution	±	-	++++	-	-	-	-	-
MP3A10	GSKPRI	Bovine kidney HS 10 min elution	+++	±	++++	-	-	-	-	-
MP4A4	GRRPRV	Bovine kidney HS 10 min elution	±	-	+++	-	-	-	-	-
MP4C6	GRRNHV	Bovine kidney HS 10 min elution	++	+	+++	-	-	-	-	-
MP4H8	GRQPRV	Bovine kidney HS 10 min elution	++	±	++++	-	-	-	-	-
MP4A11	GRQPKR	Bovine kidney HS 10 min elution	+	-	+++	-	-	-	-	-
MP3B2	GRKPRV	Bovine kidney HS 10 min elution	++	-	++++	-	-	-	-	-
MP3D2	GQRPRA	Bovine kidney HS 10 min elution	++	-	+++	-	-	-	-	-

MP3D9	GQRKRT	Bovine kidney HS 10 min elution	++	±	++++	-	-	-	-	-
MP3G2	GQKPRH	Bovine kidney HS 10 min elution	++	±	++++	-	-	-	-	-
MP3E7	GQHPRR	Bovine kidney HS 10 min elution	++	±	+++	±	-	-	-	-
MP3F1	GKRPRS	Bovine kidney HS 10 min elution	±	-	+++	-	-	-	-	-
MP3G12	GKQPRA	Bovine kidney HS 10 min elution	++	-	++++	-	-	-	±	-
MP3D6	GHSPRL	Bovine kidney HS 10 min elution	++	±	+++	-	-	-	-	-
MP3B3	GHKPRH	Bovine kidney HS 10 min elution	+++	+	++++	-	-	-	-	-
MP3E5	WRQRQA	Bovine kidney HS 20 min elution	+	+	+++	-	-	-	-	-
MP4E8	VRRSRT	Bovine kidney HS 20 min elution	-	±	++	-	-	-	-	-
MP4H12	TRRPKT	Bovine kidney HS 20 min elution	+	+	++++	±	-	-	-	-
MP4B11	TRKPRR	Bovine kidney HS 20 min elution	++	+	+++	-	-	-	-	-
MP3C2	TKHQKR	Bovine kidney HS 20 min elution	++	++	++++	±	-	-	-	-
MP4B9	SRNTKA	Bovine kidney HS 20 min elution	-	-	+	-	-	-	-	-
MP4G9	RRQPRT	Bovine kidney HS 20 min elution	±	±	+++	-	-	-	-	-
MP3B2	NRRHRT	Bovine kidney HS 20 min elution	++	±	+	-	-	-	-	-
MP4B12	NRRARQ	Bovine kidney HS 20 min elution	-	-	+	-	-	-	-	-
MP3B4	NRKHNR	Bovine kidney HS 20 min elution	++	++	++++	-	-	-	-	-
MP4E10	MRRSNP	Bovine kidney HS 20 min elution	++	+	++++	-	-	-	-	-
MP4A11	MRRLRP	Bovine kidney HS 20 min elution	+	++	+++	-	-	-	-	-
MP4E11	MRRAQP	Bovine kidney HS 20 min elution	+	+	+++	-	-	-	-	-

MP4H10	LRRHHN	Bovine kidney HS 20 min elution	++	++	++++	+	±	±	-	-
MP3E1	LRNPRN	Bovine kidney HS 20 min elution	+++	+++	+++++	-	-	-	-	-
MP3E6	IRSKDQ	Bovine kidney HS 20 min elution	-	-	±	-	-	-	-	-
MP3H4	IRRLSS	Bovine kidney HS 20 min elution	±	±	+++	-	-	-	-	-
MP3D6	IRKIKK	Bovine kidney HS 20 min elution	±	±	+++	-	-	-	-	-
MP3C6	IRHART	Bovine kidney HS 20 min elution	±	±	++	-	-	-	-	-
MP3C3	IKRLHS	Bovine kidney HS 20 min elution	++	++	+++++	-	-	-	-	-
MP4H7	GRRPRT	Bovine kidney HS 20 min elution	+	+	++++	-	-	-	-	-
MP4E7	GRRPRF	Bovine kidney HS 20 min elution	-	-	+	-	-	-	-	-
MP4F9	GRRPKR	Bovine kidney HS 20 min elution	-	-	++	-	-	-	-	-
MP4F11	GRRLRK	Bovine kidney HS 20 min elution	++	++	++++	+	-	-	-	-
MP3G1	GRKPRR	Bovine kidney HS 20 min elution	+++	+++	++++	-	-	-	-	-
MP4A9	GQRLRR	Bovine kidney HS 20 min elution	-	-	+++	-	-	-	-	-
MP4C11	GQKSRK	Bovine kidney HS 20 min elution	-	-	++++	-	-	-	-	-
MP4A7	ERRPKL	Bovine kidney HS 20 min elution	+++	+++	++++	±	-	-	-	-
MP4G11	ERRHRT	Bovine kidney HS 20 min elution	+	+	++++	±	-	-	-	-
MP3G2	ARHIQT	Bovine kidney HS 20 min elution	-	±	+	-	-	-	-	-
MP3D9	LRRAQP	HS from intestinal mucosa	++	±	+++	-	-	-	-	-
MP3A11	GSRLRR	HS from intestinal mucosa	+++	+	++++	±	-	-	-	-
MP4H8	GRKPRT	HS from intestinal mucosa	+++	++	++++	-	-	-	-	-

MP4G5	GRKPRL	HS from intestinal mucosa	+++	+	++++	-	-	-	-	-
MP3A4	GKSPRK	HS from intestinal mucosa	+	-	+++	-	-	-	-	-
MP3G7	GKQPRK	HS from intestinal mucosa	+++	+	++++	-	-	-	-	-
MP3B9	GKKPRR	HS from intestinal mucosa	++++	+++	++++	±	-	-	-	-
MP3H3	GRRHSA	Lung HS	++	+	++	-	-	-	-	-
MP4A9	GQRMKH	Lung HS	++	+	+++	±	-	-	-	-
MP3F5	GQRHSA	Lung HS	+	±	+	-	-	-	-	-
MP3D6	GNRLRS	Lung HS	+	+	++	-	-	-	-	-
MP3A5	GNQQRH	Lung HS	+	±	+	-	-	-	-	-

Reactivity: ++: strong, +: moderate, ±: weak, - absent
HS b.k.: heparan sulfate from bovine kidney; HS i.m.: heparan sulfate from porcine intestinal mucosa; Hep: heparin; DS: dermatan sulfate; CSA: chondroitin sulfate A; CSC: chondroitin sulfate C; DNA: deoxyribonucleic acid; HYA: hyaluronic acid.

Supplementary Table 5: Reactivity of anti-HS scFv antibodies against desulfated heparins as assessed by ELISA

ID	CDR3	Panning material	heparin	N-desulfated / N-acetylated heparin	2-*O* desulfated heparin	6-*O* desulfated heparin
MP4F5	TRRLKS	Bovine kidney HS 10 min elution	+	-	-	-
MP4F10	SRRHRS	Bovine kidney HS 10 min elution	±	-	-	-
MP3C1	SRRHKN	Bovine kidney HS 10 min elution	+	-	-	±
MP3H1	RRNPKL	Bovine kidney HS 10 min elution	-	-	++	++
MP3C8	RRHPQR	Bovine kidney HS 10 min elution	++	-	-	+
MP4B9	NRRSKT	Bovine kidney HS 10 min elution	++	-	-	++
MP3D5	MRKTHT	Bovine kidney HS 10 min elution	++	++	++	++
MP3H10	IRSLKP	Bovine kidney HS 10 min elution	++	+	-	++
MP3C2	GSKQRV	Bovine kidney HS 10 min elution	±	-	-	-
MP3G6	GSKPRR	Bovine kidney HS 10 min elution	++	-	-	+
MP4G3	GSKPRM	Bovine kidney HS 10 min elution	+	-	-	+
MP3A10	GSKPRI	Bovine kidney HS 10 min elution	++	-	-	±
MP4A4	GRRPRV	Bovine kidney HS 10 min elution	++	-	-	+
MP4C6	GRRNHV	Bovine kidney HS 10 min elution	±	-	-	±
MP4H8	GRQPRV	Bovine kidney HS 10 min elution	+	-	±	+
MP4A11	GRQPKR	Bovine kidney HS 10 min elution	+	-	+	+
MP3B2	GRKPRV	Bovine kidney HS 10 min elution	++	-	-	+
MP3D2	GQRPRA	Bovine kidney HS 10 min elution	+	-	-	±
MP3D9	GQRKRT	Bovine kidney HS 10 min elution	+	-	-	±
MP3G2	GQKPRH	Bovine kidney HS 10 min elution	±	-	-	-
MP3E7	GQHPRR	Bovine kidney HS 10 min elution	-	-	-	-
MP3F1	GKRPRS	Bovine kidney HS 10 min elution	ND	ND	ND	ND
MP3G12	GKQPRA	Bovine kidney HS 10 min elution	+	-	-	+
MP3D6	GHSPRL	Bovine kidney HS 10 min elution	-	-	-	-
MP3B3	GHKPRH	Bovine kidney HS 10 min elution	++	-	-	++
MP3E5	WRQRQA	Bovine kidney HS 20 min elution	+	±	+	-

MP4E8'	VRRSRT	Bovine kidney HS 20 min elution	++	-	-	-
MP4H12	TRRPKT	Bovine kidney HS 20 min elution	++	-	-	++
MP4B11	TRKPRR	Bovine kidney HS 20 min elution	++	-	-	-
MP3C2	TKHQKR	Bovine kidney HS 20 min elution	++	+	++	++
MP4B9	SRNTKA	Bovine kidney HS 20 min elution	-	-	-	-
MP4G9	RRQPRT	Bovine kidney HS 20 min elution	+	-	-	+
MP3B2	NRRHRT	Bovine kidney HS 20 min elution	++	-	-	-
MP4B12	NRRARQ	Bovine kidney HS 20 min elution	++	+	+	+
MP3B4	NRKHNR	Bovine kidney HS 20 min elution	++	-	-	+
MP4E10	MRRSNP	Bovine kidney HS 20 min elution	++	±	+	+
MP4A11	MRRLRP	Bovine kidney HS 20 min elution	++	-	+	++
MP4E11	MRRAQP	Bovine kidney HS 20 min elution	+	+	±	-
MP4H10	LRRHHN	Bovine kidney HS 20 min elution	++	++	++	++
MP3E1	LRNPRN	Bovine kidney HS 20 min elution	++	+	±	++
MP3E6	IRSKDQ	Bovine kidney HS 20 min elution	-	-	-	-
MP3H4	IRRLSS	Bovine kidney HS 20 min elution	++	+	++	++
MP3D6	IRKIKK	Bovine kidney HS 20 min elution	±	-	-	-
MP3C6	IRHART	Bovine kidney HS 20 min elution	+	-	-	-
MP3C3	IKRLHS	Bovine kidney HS 20 min elution	++	±	±	+
MP4H7	GRRPRT	Bovine kidney HS 20 min elution	++	-	-	+
MP4E7	GRRPRF	Bovine kidney HS 20 min elution	++	-	-	+
MP4F9	GRRPKR	Bovine kidney HS 20 min elution	++	-	±	+
MP4F11	GRRLRK	Bovine kidney HS 20 min elution	++	+	++	++
MP3G1	GRKPRR	Bovine kidney HS 20 min elution	++	-	-	++
MP4A9	GQRLRR	Bovine kidney HS 20 min elution	++	+	+	++
MP4C11	GQKSRK	Bovine kidney HS 20 min elution	++	-	-	+
MP4A7	ERRPKL	Bovine kidney HS 20 min elution	++	-	+	++
MP4G11	ERRHRT	Bovine kidney HS 20 min elution	+	-	-	±
MP3G2	ARHIQT	Bovine kidney HS 20 min elution	-	-	-	-
MP3D9	LRRAQP	HS from intestinal mucosa	++	+	++	++

MP3A11	GSRLRR	HS from intestinal mucosa	++	+	-	-
MP4H8	GRKPRT	HS from intestinal mucosa	++	±	+	++
MP4G5	GRKPRL	HS from intestinal mucosa	++	-	-	++
MP3A4	GKSPRK	HS from intestinal mucosa	++	-	-	-
MP3G7	GKQPRK	HS from intestinal mucosa	++	+	+	++
MP3B9	GKKPRR	HS from intestinal mucosa	++	-	±	++
MP3H3	GRRHSA	Lung HS	++	+	+	++
MP4A9	GQRMKH	Lung HS	++	++	+	++
MP3F5	GQRHSA	Lung HS	±	-	-	++
MP3D6	GNRLRS	Lung HS	++	-	±	++
MP3A5	GNQQRH	Lung HS	+	-	-	++

Reactivity: ++: strong, +: moderate, ±: weak, - absent.
ND: No data

Supplementary Table 6: Distribution of HS epitopes defined by all anti-HS antibodies in rat kidney

ID	CDR3	Panning material	Glomerular capillary tuft	Bowman's capsule	Mesangium	Tubuli	Peritubular capillaries	Blood vessels
MP4F5	TRRLKS	Bovine kidney HS 10 min elution	+++	-	ND	-	±	++
MP4F10	SRRHRS	Bovine kidney HS 10 min elution	+++	-	ND	-	±	++
MP3C1	SRRHKN	Bovine kidney HS 10 min elution	++	-	+	ND	+	++
MP3H1	RRNPKL	Bovine kidney HS 10 min elution	+++	-	ND	+	+	+
MP3C8	RRHPQR	Bovine kidney HS 10 min elution	++	+	++	+	+	+
MP4B9	NRRSKT	Bovine kidney HS 10 min elution	+	±	++	±	+	±
B MP3D5	MRKTHT	Bovine kidney HS 10 min elution	++	-	+	-	+	+
MP3H10	IRSLKP	Bovine kidney HS 10 min elution	+	++	+	±	+	-
MP3C2	GSKQRV	Bovine kidney HS 10 min elution	-	-	-	-	-	-
MP3G6	GSKPRR	Bovine kidney HS 10 min elution	+	++	±	±	+	±
MP4G3	GSKPRM	Bovine kidney HS 10 min elution	+	++	ND	-	+	-
MP3A10	GSKPRI	Bovine kidney HS 10 min elution	+	++	+	±	+	-
MP4A4	GRRPRV	Bovine kidney HS 10 min elution	+	++	ND	+	+	+
MP4C6	GRRNHV	Bovine kidney HS 10 min elution	++	-	+	-	+	+
MP4H8	GRQPRV	Bovine kidney HS 10 min elution	+	++	±	±	+	-
MP4A11	GRQPKR	Bovine kidney HS 10 min elution	-	++	-	±	±	-
MP3B2	GRKPRV	Bovine kidney HS 10 min elution	+	++	ND	+	+	+
MP3D2	GQRPRA	Bovine kidney HS 10 min elution	±	-	ND	ND	+	±
MP3D9	GQRKRT	Bovine kidney HS 10 min elution	±	+	ND	-	+	-
MP3G2	GQKPRH	Bovine kidney HS 10 min elution	±	±	+	-	+	+
MP3E7	GQHPRR	Bovine kidney HS 10 min elution	-	-	-	-	-	-
MP3F1	GKRPRS	Bovine kidney HS 10 min elution	ND	ND	ND	ND	ND	ND
MP3G12	GKQPRA	Bovine kidney HS 10 min elution	±	+	+	-	+	-
MP3D6	GHSPRL	Bovine kidney HS 10 min elution	-	-	-	-	-	-
MP3B3	GHKPRH	Bovine kidney HS 10 min elution	+	±	+	±	+	±
MP3E5	WRQRQA	Bovine kidney HS 20 min elution	±	±	+	-	±	ND
MP4E8'	VRRSRT	Bovine kidney HS 20 min elution	++	-	+	-	+	+

MP4H12	TRRPKT	Bovine kidney HS 20 min elution	++	-	+	-	+	+
MP4B11	TRKPRR	Bovine kidney HS 20 min elution	±	+	+	±	+	ND
MP3C2	TKHQKR	Bovine kidney HS 20 min elution	++	-	+	-	+	+
MP4B9	SRNTKA	Bovine kidney HS 20 min elution	-	-	-	-	-	-
MP4G9	RRQPRT	Bovine kidney HS 20 min elution	±	±	±	±	+	+
MP3B2	NRRHRT	Bovine kidney HS 20 min elution	++	-	ND	-	+	+
MP4B12	NRRARQ	Bovine kidney HS 20 min elution	++	-	ND	-	+	ND
MP3B4	NRKHNR	Bovine kidney HS 20 min elution	++	-	+	-	+	+
MP4E10	MRRSNP	Bovine kidney HS 20 min elution	±	+++	+	±	+	ND
MP4A11	MRRLRP	Bovine kidney HS 20 min elution	±	+	+	-	+	-
MP4E11	MRRAQP	Bovine kidney HS 20 min elution	+++	-	ND	-	±	++
MP4H10	LRRHHN	Bovine kidney HS 20 min elution	±	+	+	±	+	ND
MP3E1	LRNPRN	Bovine kidney HS 20 min elution	±	+++	+	±	+	ND
MP3E6	IRSKDQ	Bovine kidney HS 20 min elution	-	-	-	-	-	-
MP3H4	IRRLSS	Bovine kidney HS 20 min elution	±	+	+	-	+	-
MP3D6	IRKIKK	Bovine kidney HS 20 min elution	-	-	-	-	-	-
MP3C6	IRHART	Bovine kidney HS 20 min elution	++	-	+	-	+	+
MP3C3	IKRLHS	Bovine kidney HS 20 min elution	±	±	++	-	+	-
MP4H7	GRRPRT	Bovine kidney HS 20 min elution	++	-	+	-	+	+
MP4E7	GRRPRF	Bovine kidney HS 20 min elution	++	-	+	-	+	+
MP4F9	GRRPKR	Bovine kidney HS 20 min elution	++	-	+	-	+	+
MP4F11	GRRLRK	Bovine kidney HS 20 min elution	++	+	+	-	+	+
MP3G1	GRKPRR	Bovine kidney HS 20 min elution	++	-	+	-	+	+
MP4A9	GQRLRR	Bovine kidney HS 20 min elution	++	-	+	-	+	+
MP4C11	GQKSRK	Bovine kidney HS 20 min elution	-	+	±	±	+	+
MP4A7	ERRPKL	Bovine kidney HS 20 min elution	++	-	+	-	+	+
MP4G11	ERRHRT	Bovine kidney HS 20 min elution	++	-	+	-	+	+
MP3G2	ARHIQT	Bovine kidney HS 20 min elution	++	-	+	-	+	+
MP3D9	LRRAQP	HS from intestinal mucosa	++	-	+	-	+	+
MP3A11	GSRLRR	HS from intestinal mucosa	++	-	+	-	+	+

MP4H8	GRKPRT	HS from intestinal mucosa	++	-	+	-	+	+
MP4G5	GRKPRL	HS from intestinal mucosa	+	+	+	-	+	+
MP3A4	GKSPRK	HS from intestinal mucosa	±	++	±	-	±	ND
MP3G7	GKQPRK	HS from intestinal mucosa	±	++	+	-	+	+
MP3B9	GKKPRR	HS from intestinal mucosa	++	-	+	-	+	+
MP3H3	GRRHSA	Lung HS	±	++	±	-	±	ND
MP4A9	GQRMKH	Lung HS	±	++	±	-	±	ND
MP3F5	GQRHSA	Lung HS	±	++	±	-	±	ND
MP3D6	GNRLRS	Lung HS	±	++	±	-	±	ND
MP3A5	GNQQRH	Lung HS	++	-	±	-	±	ND

Staining: ++: strong, +: moderate, ±: weak, - absent. Blanks indicate the absence of the morphological structure or uncertainty of staining intensity.
ND: No data
