# Supplementary material for: Construction and evaluation of an antibody phage display library targeting heparan sulfate
Source: Glycoconj J. 2020 May 28;37(4):445–55. doi: 10.1007/s10719-020-09925-z (PMC7329785; doi:10.1007/s10719-020-09925-z)
Supplement: Supplementary file 2 — (DOCX 12.2 kb) [file 10719_2020_9925_MOESM2_ESM.docx]

**Supplementary Table 2:** Composition of modified heparin molecules (taken from [31]).

| **Molecule** | **Characteristics** |
| --- | --- |
| Heparin | 11.3% N-acetyl, 88.7% N-sulfate, 69% 2-*O* sulfate, 79% 6-*O* sulfate |
| N-desulfated/N-acetylated heparin | 100% N-acetyl, 0% N-sulfate, 69% 2-*O* sulfate, 79% 6-*O* sulfate |
| 2-*O* desulfated heparin | 13% N-acetyl, 87% N-sulfate, 0% 2-*O* sulfate, 79% 6-*O* sulfate |
| 6-*O* desulfated heparin | 13% N-acetyl, 87% N-sulfate, 67% 2-*O* sulfate, 23% 6-*O* sulfate |
